# Physical Human Activity Recognition Using Wearable Sensors

**DOI:** 10.3390/s151229858

**Published:** 2015-12-11

**Authors:** Ferhat Attal, Samer Mohammed, Mariam Dedabrishvili, Faicel Chamroukhi, Latifa Oukhellou, Yacine Amirat

**Affiliations:** 1 Laboratory of Images, Signals and Intelligent Systems (LISSI), University of Paris-Est Créteil (UPEC), 122 rue Paul Armangot, Vitry-Sur-Seine 94400, France; ferhat.attal@u-pec.fr (F.A.); mariam.dedabrishvili@univ-paris-est.fr (M.D.); amirat@u-pec.fr (Y.A.); 2 Laboratory of Information Science and Systems (LSIS, CNRS-UMR7296), University of Toulon, Bâtiment R, BP 20132, La Garde Cedex 83957, France; faicel.chamroukhi@univ-tln.fr; 3 French Institute of Science and Technology for Transport, development and Networks (IFSTTAR), University of Paris-Est, COSYS, GRETTIA, Marne la Vallée F-77447, France; latifa.oukhellou@ifsttar.fr

**Keywords:** activity recognition, wearable sensors, smart spaces, data classifiers, accelerometers, physical activities

## Abstract

This paper presents a review of different classification techniques used to recognize human activities from wearable inertial sensor data. Three inertial sensor units were used in this study and were worn by healthy subjects at key points of upper/lower body limbs (chest, right thigh and left ankle). Three main steps describe the activity recognition process: sensors’ placement, data pre-processing and data classification. Four supervised classification techniques namely, k-Nearest Neighbor (k-NN), Support Vector Machines (SVM), Gaussian Mixture Models (GMM), and Random Forest (RF) as well as three unsupervised classification techniques namely, k-Means, Gaussian mixture models (GMM) and Hidden Markov Model (HMM), are compared in terms of correct classification rate, F-measure, recall, precision, and specificity. Raw data and extracted features are used separately as inputs of each classifier. The feature selection is performed using a wrapper approach based on the RF algorithm. Based on our experiments, the results obtained show that the k-NN classifier provides the best performance compared to other supervised classification algorithms, whereas the HMM classifier is the one that gives the best results among unsupervised classification algorithms. This comparison highlights which approach gives better performance in both supervised and unsupervised contexts. It should be noted that the obtained results are limited to the context of this study, which concerns the classification of the main daily living human activities using three wearable accelerometers placed at the chest, right shank and left ankle of the subject.

## 1. Introduction

The aging population is constantly increasing around the world. In the last decade, the active involvement and participation of the elderly in society became an important challenge from a social and economic point of view. Currently, assisting elderly people during their daily activities, increasing their safety, well-being and autonomy, are considered key research challenges of great interest. Activity recognition based on new wearable technologies (wearable sensors and accessories, smartphones, *etc*.) is one of these important challenges. Recognizing and monitoring human activities are fundamental functions to provide healthcare and assistance services to elderly people living alone, physically or mentally disabled people, and children. These populations need continuous monitoring of their activities to detect abnormal situations or prevent unpredictable events such as falls [1]. The new technologies of health monitoring devices range from on-body wearable sensors to *in vivo* sensors. For instance, bio-sensors are generally used to monitor vital signs such as electrocardiography (ECG), electromyography (EMG), blood pressure, heart rate and temperature [2]. Illnesses such as seizures, hypertension, dysthymias, and asthma can be diagnosed and treated by physiological monitoring. Inclinometers and goniometers are other types of sensors that are used to measure upper/lower limbs kinematics [3]. Even though there are potential gains of a remote monitoring system using wearable sensors, there are still challenges in terms of technological advancements to design wearable sensors that are easy to use and comfortable for the wearer [4]. Continuous reduction in the size and energy consumption of these sensors is another challenge that needs to be addressed. 

**Figure 1:**
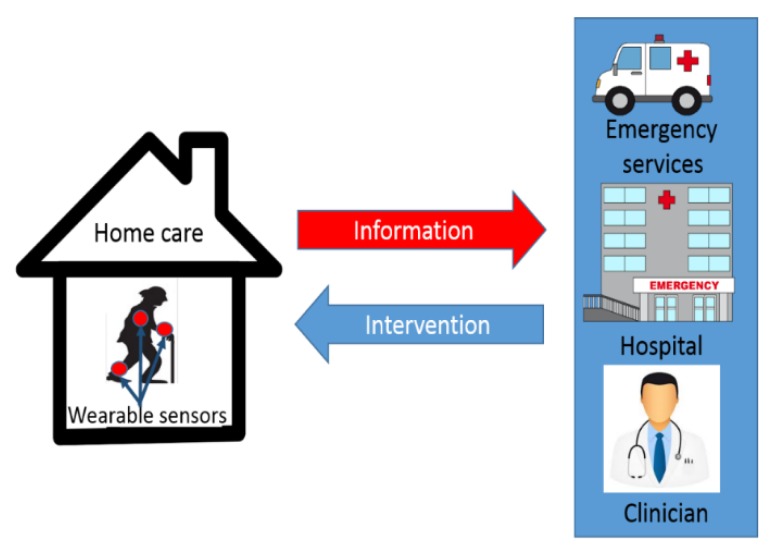
Remote health monitoring architecture based on wearable sensors.

This paper focuses on a review of human activities recognition using wearable sensors in the context of the remote monitoring of an elderly or dependent subject (Figure 1). The home supportive environment delivers trend data and detection of incidents using non-intrusive wearable sensors. This facilitates a quick measurement and fast acceptance at the same time. Through real-time processing and data transmission, healthcare suppliers will be able to monitor the subject’s motions during daily activities and also to detect unpredictable events that may occur, like a fall [1]. The subject’s records can be used in medical decision support, prediction and prevention.

Inertial sensors have been used mainly for navigation of aircraft, ships, land vehicles and robots, and also for shock and vibration analysis in several industries. Rapid development of micro-electromechanical systems technology has contributed to the development of small-size, light-weight and low-cost inertial sensors [5]. Currently, many manufacturers propose inertial sensors that are easy to attach and wear. These sensors allow one to collect data on daily living activities under free-living conditions and over extended periods of time. The number and the placement of inertial sensors on the human body have a direct impact on activity recognition, in terms of the variety of activities to monitor and the precision of their classification. Vision-based systems using single or multiple video cameras are also used to recognize daily living activities. These systems are suitable for motion capture when activities are mainly achieved in small areas, such as an office or house environment. The most popular system is the Kinect sensor released recently by Microsoft. It includes an infrared depth-finding camera and a standard RGB camera. This sensor has several advantages such as low cost, depth information and the ability to operate any time, even at night. However, it has low performance in natural lighting conditions, causing a shadowing of the points of interest [6]. The inability to record moving objects at a long distance along with the dependence on surface texturing and the occlusion problem in cluttered environments represent other disadvantages of the Kinect. In addition, the computational and storage costs incurred by image processing are relatively high compared to wearable inertial sensors.

This paper deals with the classification of daily living human activities using wearable inertial sensors. Walking, lying, standing up, *etc*. are examples of these activities. In this study, a dataset is collected using three inertial sensors and including 12 daily living activities, namely: standing, stair descent, sitting, sitting down, sitting on the ground, from sitting to sitting on the ground, from lying to sitting on the ground, lying down, lying, walking, stair ascent and standing up. The choice of these selected activities was made to represent the majority of everyday living activities [7]. In addition to these basic activities, emphasis has also been placed on the dynamic transitions between those activities, which constitute phases where the elderly are more vulnerable and potentially exposed to falls such as sitting down, standing up, from sitting to sitting on the ground. Most of the selected activities were used in recent studies related to the recognition of human activities with elderly subjects [8,9]. Four supervised classification techniques namely, k-Nearest Neighbor (k-NN), Support Vector Machines (SVM), Supervised Learning Gaussian Mixture Models (SLGMM) and Random Forest (RF) as well as three unsupervised classification techniques namely, k-Means, Gaussian Mixture Models (GMM) and Hidden Markov Model (HMM), are compared in terms of correct classification rate, F-measure, recall, precision, and specificity. The main objective of implementing several classification techniques is to review, compare and evaluate their performance using a real dataset. Raw data and extracted features are used separately as inputs of each classifier. The inertial sensor units worn by different healthy subjects were placed at key points of upper/lower body limbs (chest, right thigh and left ankle). The activity recognition process includes three main steps: sensors’ placement, data pre-processing and data classification. Unlike other recent research works done in the same context of this study, only acceleration data are used, in this paper as a modality for estimating the activities [5,10]. Moreover, the results obtained with unsupervised classification algorithms are provided and analyzed.

This paper in organized as follows: in Section 2, background on wearable sensor’s placement, pre-processing data including feature extraction/selection and classification techniques used in the field of human activity recognition, are addressed. The adopted methodology including data acquisition process, use of different classifiers in supervised/unsupervised contexts and performance evaluation, are presented in Section 3. Section 4 presents the experimental results obtained using a real dataset (raw data and extracted/selected features). Finally, a conclusion and some research perspectives are given in Section 5.

## 2. Backgrounds on Sensors’ Placement, Data Pre-Processing and Classification Techniques

In this section, we present a background on wearable sensor’s placement, pre-processing data including feature extraction and selection and classification techniques used in the field of human activity recognition.

### 2.1. Wearable Sensors’ Placement

**Figure 2:**
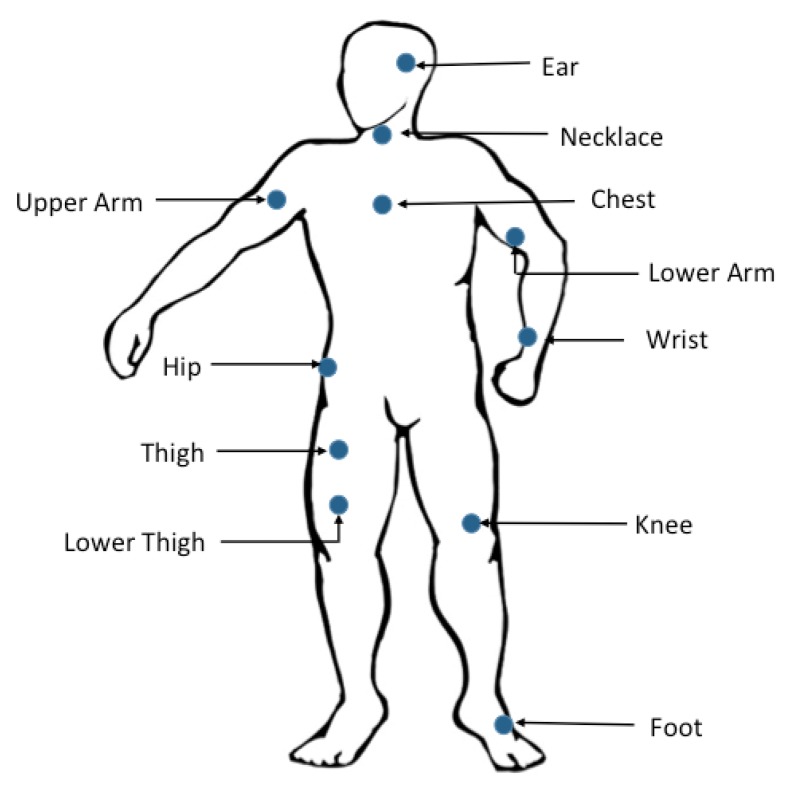
Graphical illustration of wearable sensor placement.

The placement of wearable sensors is related to the locations where the sensors are placed and how they are attached to those locations. Indeed, wearable sensors placement has a direct effect on the measurement of bodily motions [11], but the ideal sensor location for particular applications is still a subject of much debate [12]. As shown in Figure 2, wearable sensors can be placed on different parts of the human body. In particular, the sensors are usually placed on the sternum [13], lower back [14], and waist [15]. Waist-placement of the wearable sensors can better represent most human motions since they are then close to the center of mass of the human body [16].

Various studies have combined multiple accelerometers attached at different locations of the body (see Table 1). The majority of these studies highlight that the placement of many sensors can become burdensome for the wearer, leading us to focus on determining both the minimum number of sensors as well as their relevant placement, while still ensuring a sufficiently high activity recognition rate. Indeed, this rate decreases with the number of wearable accelerometers. As observed in Table 1, accuracy levels of 83% to 100% for human activity recognition rates have been obtained [14,17,18,19]. In [19] an accuracy of 100% was obtained for recognition of some activities such as sitting, lying, standing and walking across a series of 40 randomly chosen tasks. This result is somehow flawed as it is obtained on very simple activities, while performance with complex activities was not evaluated. 

**Table 1:** Review of studies on accelerometer placement for human activity recognition.

Reference	Placement ofAccelerometers	Detected Activities	Average (%) ofClassification Accuracy
Karantonis *et al.*, 2006 [15]	Waist	Walking, Falling	90.8%
Mathie, 2004 [18]	Waist	Falling, Walking, Sitting, Standing, Lying	98.9%
Yang *et al.*, 2008 [20]	Wrist	Walking, Running, Scrubbing, Standing, Working at a PC, Vacuuming, Brushing teeth Sitting	95%
Pirttikangas, 2006 [21]	Thigh, Necklace, Wrists	Typing, Watching TV, Drinking, Stairs Ascent and Descent	91.5%
Parkka, 2006 [17]	Wrist, Chest	Lying, Sitting, Walking, Rowing And Cycling	83.3%
Olguın, 2006 [22]	Wrist, Chest, Hip	Sitting, Running, Walking, Standing, Lying, Crawling	92.13%
Bonomi, 2009 [14]	Lower Back	Lying, Sitting, Standing, Working on a Computer, Walking, Running, Cycling	93%
Yeoh, 2008 [19]	Thigh, Waist	Sitting, Lying, Standing And Walking Speed	100%
Lyons, 2005 [23]	Thigh, Trunk	Sitting, Standing, Lying, Moving	92.25%
Salarian *et al.*, 2007 [24]	Trunk , shanks (IMU sensor)	14 daily living activities	-
Gjoreski, 2011 [25]	Thigh, Waist, Chest, Ankle	Lying, Sitting, Standing, All Fours, Transitional	91%
Chamroukhi, 2013 [7]	Chest, Thigh, Ankle	Stairs Ascent and Descent, Walking, Sitting, Standing Up, Sitting on the Ground	90.3%
Bayat *et al.*, 2014 [26]	pocket, Hand	Slow Walking, Fast Walking, Running, Stairs-Up, Stairs-Down, and Dancing	91.15%
Moncada-Torres, 2014 [27]	Chest, Thigh, Ankle	16 daily living activities	89.08%
Gupta *et al.* 2014 [28]	Waist	walking, jumping, running, sit-to-stand/stand-to-sit, stand-to-kneel-to-stand, and being stationary	98%
Garcia-Ceja *et al.*, 2014 [29]	Wrist	long-term activities (Shopping, Showering, Dinner, Working, Commuting, Brush Teeth)	98%
Gao *et al.*, 2014 [8]	Chest, waist, thigh, side	standing, sitting, lying, walking and transition	96.4%
Massé *et al.* [30]	Trunk (IMU and barometric pressure sensor)	sitting, standing, walking, lying	90.4%

Cleland *et al.* [12] reported their investigations on everyday activities such as walking, jogging on a motorized treadmill, sitting, lying, standing, stairs ascent and descent. The data were obtained from six sensors placed on different locations on the body (the chest, left hip, left wrist, left thigh, left foot and lower back). The results obtained in this study showed that the sensor placed on the hip provides the best measures to recognize most everyday activities.

Other researchers have investigated the optimal placement of the accelerometers for human activity recognition. Gjoreski *et al.* [25] studied the optimal location of accelerometers for fall detection. Four accelerometers were placed at the chest, waist, ankle and thigh. The authors indicated that the best accuracy rate was achieved by combining sensors placed at the chest or the waist and the ankle. Chamroukhi *et al.* [7] have also evaluated the impact of the number of the sensors and their location on the accuracy of human activity recognition. The best results were obtained for a configuration with three sensors located on the chest, thigh and ankle. These results demonstrated that the human activity recognition could be significantly improved by combining accelerometers located on both the upper and lower parts of the body. 

According to Karantonis [15], Mathie [18], Parkka [17] and Yang [20] data acquired from a sensor placed on the waist gives pertinent information about many activities such as sitting, standing, walking, lying in various positions, running, stairs ascent and descent, vacuuming and scrubbing. Other accelerometer placement locations such as on the wrist, chest, hip, lower back, thigh and trunk have also been used to identify lying, sitting, walking, running, cycling, working on a computer, *etc.* [14,19,22,23]. As for recognition of typing, watching TV, drinking, stairs ascent and descent, Pirttikangas *et al.* [21] used the wrists, thigh and necklace as relevant sensor placement locations on the body. 

Raj *et al.* [31] classified human daily activities such as walking, running, stairs ascent/descent, or driving a vehicle using a watch with an embedded tri-axial accelerometer. Wrist-worn accelerometers can also be used to estimate sleep duration and activity levels during sleep [32]. Ankle-attached accelerometers are able to efficiently estimate steps, travel distance, velocity and energy expenditure [17,33]. Accelerometers placed at the top of the head have been also used for measuring balance during walking [34].

In [8] the authors evaluated the accelerometer based multi-sensor *versus* single-sensors in activity recognition. Performances of six representative wearable systems with single sensors or multiple sensors were compared. The authors showed that the multi-sensor system gives the highest recognition rate. In [30], an event-based activity classifier is proposed to monitor and recognize daily living activities in mobility-impaired stroke patients using a trunk-fixed sensor that integrates barometric pressure (BP) and inertial sensors. The authors proposed a double-stage hierarchical fuzzy logic inference system. The first stage processed the events such as the start/end of lying or walking periods, and detected postural transitions while the second stage improved the activity recognition by providing a simple way to integrate the typical behavior of the subject and biomechanical constraints. 

Sensor attachment to the human body involves fixing sensors directly to skin [13] as well as using an assortment of straps, belts and wristbands [34,35]. Wearable devices can also be integrated into clothing. In order to avoid relative motion between the sensors and the human body, the wearable sensors should be correctly attached to the human body. Otherwise, the vibration or displacement of those sensors may cause signal interference and thus deterioration of the measurement accuracy.

New technological advancements and the invasion of smartphones in our daily lives offer new opportunities for daily living human activities research. Recently, many systems have been proposed to recognize daily living human activities using data acquired from mobile phones [26,36]. Accelerometer data collected with a wrist-watch was used by Garcia-Ceja *et al.* [29] to segment long-term activity. An overview of studies according to combinations of sensors placement for human activity recognition is given in Table 1.

Although the type, the number and the placement of the sensors are important for ensuring relatively high rates of human activities recognition, issues related to acceptance of such kind of sensors and wearer’s privacy should also be taken into account. These aspects are addressed in part in [37], where the authors investigated different types of monitoring technologies for in-home activiy monitoring and their effects on the wearer.

### 2.2. Pre-Processing

Data pre-processing is one of the most important steps in the data mining process. It consists of filtering data, replacing the missing and outlier’s values and extracting/selecting features. To extract features from raw data, windowing techniques are generally used, which consist of dividing sensor signals into small time segments. Segmentation and classification algorithms are then applied respectively to each window. Three types of windowing techniques are usually used: (i) sliding window where signals are divided into fixed-length windows; (ii) event-defined windows, where pre-processing is necessary to locate specific events, which are further used to define successive data partitioning and (iii) activity-defined windows where data partitioning is based on the detection of activity changes. The sliding window approach is well-suited to real-time applications since it does not require any pre-processing treatments [3]. 

#### 2.2.1. Features Computation

Human activity recognition from inertial data is generally preceded by a feature extraction step. Signal characteristics such as time-domain and frequency-domain features are widely used for feature calculation. Time-domain features include mean, median, variance, skewness, kurtosis, range, *etc*. Peak frequency, peak power, spectral power on different frequency bands and spectral entropy are generally included in the frequency-domain features. Some of the common time-domain and frequency-domain features used for human activity recognition are presented in the following:

- Time-domain features

Time-domain features include mean, median, variance, skewness, kurtosis, range, *etc*. These features are widely used in the field of human activity recognition [5,38,39]. Bouten *et al.* [39], applied the integral method to offer estimation of energy expenditure using an inertial sensor. The authors used the total Integral of Modulus of Accelerations (IMA). This metric is referred to the time integrals of the module of accelerometer signals (Equation (4)):

(1) $$\;IMA_{tot}=\overset{N}{\underset{t=1}{\int }}\left|a_{x}\right|dt+\overset{N}{\underset{t=0}{\int }}\left|a_{y}\right|dt+\overset{N}{\underset{t=0}{\int }}\left|a_{z}\right|dt\;$$

where $a_{x}$, $a_{y}$, $a_{z}\;$ denote the orthogonoal components of accelerations, *t* denotes time and *N* represents the window length. 

Other time-domain features such as Zero-Crossings Correlation-Coefficient root mean square, *etc*. are also used in [40].

- Frequency-domain features

Discrete Fourier Transform (DFT) is used to compute frequency spectrum of the discrete data signal *x*. The DFT is described as follows [41]:

(2) $$X\left(f\right)=\overset{N-1\;}{\underset{i=0}{\sum }}\;x_{i}\;e^{-j2\pi fi/N}\;$$

where *X* denotes the frequency spectrum, *f* the *f* *^th^* Fourier coefficient in the frequency domain and *N* the length of the sliding window. Equation (5) can be rewritten using the following form:

(3) $$X\left(f\right)=\overset{N-1}{\underset{i=0}{\sum }}a_{i}+jb_{i}$$

with $a_{i}=x_{i}\cos(\frac{2\pi fi}{N})$ and $b_{i}=x_{i}\sin(\frac{2\pi fi}{N})$.

One of the most important frequency-domain features used for human activity recognition is the Power Spectral Density (PSD). This feature has been used by [42] to recognize activities such as walking, cycling, running and driving. PSD can be computed as the squared sum of its spectral coefficients normalized by the length of the sliding window:

(4) $$P\left(f\right)=\frac{1}{N}\;\overset{N-1}{\underset{i=0}{\sum }}a_{i}^{2}+b_{i}^{2}$$

Peak frequency represents the frequency corresponding to the highest computed power spectrum density over the sliding window. The peak frequency has been used in several studies related to activity recognition [40,42,43].

The entropy is another feature that is widely used in human activity recognition [44]. Generally, this feature helps to discriminate between activities that have the same PSD but different patterns of movement [43]. Entropy can be formulated as follows:

(5) $$H\left(f\right)\;=\;\frac{1}{N}\;\overset{N-1}{\underset{i=0}{\sum }}c_{i}\text{log}\left(c_{i}\right),c_{i}\;=\;\frac{\sqrt{a_{i}^{2}+b_{i}^{2}}}{\sum_{k=0}^{N-1}\sqrt{a_{i}^{2}+b_{i}^{2}}}$$

The DC component is another important feature also used in human activity recognition [43] . It represents the PDS at frequency *f* = 0 Hz. It can be formulated as the squared sum of its real spectral coefficients normalized by the length of the sliding window:

(6) $$DC=\frac{1}{N}\;\overset{N-1}{\underset{i=0}{\sum }}a_{i}^{2}$$

Other frequency-domain features based on wavelet analysis are also used in human activity recognition. For more information the reader is referred to [40,45].

#### 2.2.2. Feature Selection

Feature selection consists of selecting a subset of relevant features from the original feature set [46]. To differentiate between samples, classification algorithms need representative features. Using inappropriate or redundant features may deteriorate the performance of a classification algorithm.

This may result in a curse of the dimensionality problem and a decrease of classifier performance, therefore, the selection of a reduced number of features, which have optimal discriminative power between classes, is significant in data mining. The feature selection process is defined as a process of searching a subset of appropriate features from the original set. Feature selection is an important step in the use of machine-learning algorithms as it reduces computation time and complexity, while improving the overall classification rate.

Liu *et al.* [47] categorized the feature selection process in a three- dimensional framework into a data mining task, an evaluation criterion, and a search strategy. The feature selection process is generally categorized into three categories: filter methods [47], wrapper methods [48] and hybrid methods [49]. Filter methods operate directly on the dataset by exploiting the intrinsic properties of the features. These methods rank a set of selected features according to the estimated weights of each feature. It should be noted that filter methods do not use any classifier in the selection process. Unlike filter methods, wrapper methods, which often yield better results, use a classifier to evaluate the selected subsets based on their predictive accuracies. Finally, the hybrid methods select the most relevant subset based on the use of some internal parameters of the machine-learning algorithm. In these methods, no validation step is required in the process of feature selection. For more details of using feature selection methods in human activity recognition application, the reader is invited to consult some related works [50,51]. 

#### 2.2.3. Feature Extraction

The combination of original features is an alternative way of selecting a subset of relevant features. This technique consists of combining the original features set in order to define a new relevant features set. In other words, feature extraction is the transformation of high-dimensional data into a meaningful representation data of reduced dimensionality. The main advantage of feature extraction is that it facilitates classification and visualization of high-dimensional data. 

The most popular technique for feature extraction is principal component analysis (PCA) [52], which is a linear technique that consists of transforming the original features (generally inter-correlated) into new mutually uncorrelated features. These new features are the so-called principal components. The main idea behind PCA is to remap the original features into a low dimensional space in which the principal components are arranged according to their variance (from largest to lowest). The principal components that contribute to very low variance are omitted. 

Linear Discriminant Analysis (LDA) also extracts features through a linear transformation. LDA is closely associated to principal component analysis (PCA) since these two methods try to find linear combinations of variables, which best represent the data [53]. The LDA method projects the original features points into a new space of lower dimension that maximizes the between-class separability while minimizing their within-class variability unlike PCA which, does not take into account any difference in classes.

The independent component analysis (ICA) [54] is another feature extraction technique commonly used on non-Gaussian data. This technique was initially developed to provide solution to a problem known as Blind Source Separation (BSS). ICA searches for projections of original features such that the probability distributions of the projected data are statistically independent. The ICA algorithm aims at finding independent components, such as the original features that can be expressed as a linear combination of those components.

Another feature extraction method used in data mining is Factors Analysis (FA). In the FA method, the original features can be grouped according to their correlation, however, FA represents each group of features that are highly correlated but have small correlations with features in other groups by some factor. For more details on using feature extraction methods in human activity recognition application, the reader is invited to refer to some related works in [50,51,55,56].

### 2.3. Classification Techniques

The features extracted/selected from the raw sensor data are used as inputs of the classification algorithms. In case of human activity recognition, the patterns of input data are associated with the activities (classes) under consideration. In general, the classification task requires learning a decision rule or a function associating the inputs data to the classes. There are two main directions in machine learning techniques: supervised and unsupervised approaches [54,57,58]. Supervised learning approaches for classification such as artificial neural networks [57], Support Vector Machines (SVM) [59], require entirely labeled activity data. The unsupervised learning approaches, such as those based on Gaussian Mixture Models (GMMs), Hidden Markov Models (HMMs) [60] allow to infer automatically the labels from the data. 

In the following sections, we briefly describe the classification techniques used in this study (GMMs, k-Nearest Neighbors (k-NN), SVMs, Random Forests (RFs), K-means and HMMs), as well as other techniques that are widely used in human activity recognition such as multilayer perceptron, naive Bayes, hierarchical classification, *etc*.

#### 2.3.1. k-Nearest Neighbors

k-Nearest Neighbors (k-NN) [54,58] is a supervised classification technique that can be seen as a direct classification method because it does not require a learning process. It just requires the storage of the whole data. To classify a new observation, the K-NN algorithm uses the principle of similarity (distance) between the training set and new observation to classify. The new observation is assigned to the most common class through a majority vote of its k nearest neighbors. The distance of the neighbors of an observation is calculated using a distance measurement called similarity function such as Euclidean distance. Moreover, one should note that when using the K-NN approach and a new sample is assigned to a class, the computation of distances (*i.e.*, the computation time) increases as a function of the existing examples in the dataset [61].

Foerster *et al.* [62] were the first to apply the k-NN classification to differentiate between nine human activities using time-domain features obtained from three uni-axial accelerometers. In [63] Foerster and Fahrenberg combined k-NN with a hierarchical decision approach to recognize nine activities using frequency-domain features. This approach has shown to be more efficient, in terms of classification accuracy, compared to the k-NN. Other studies based on k-NN for human activity recognition have also shown a high level of accuracy and satisfactory segmentation results [7,64]. 

#### 2.3.2. Support Vector Machines

Support Vector Machines (SVMs), introduced by Vapnik [59], is a classifier derived from statistical learning theory. This well-known machine learning technique that minimizes an empirical risk (as a cost function) and at the same time, maximizes the margin between the so-called separating hyperplane and the data.

In their standard formulation, SVMs are linear classifiers. However, non-linear classification can be achieved by extending SVM by using kernels methods [65]. The key idea of kernels methods is to project the data from the original data space to a high dimensional space called feature space by using a given non-linear kernel function. A linear separation in the resulting feature space can then be achieved by using the Cover’s theorem [66]. Moreover, SVM is a binary classifier; therefore, to ensure a multi-class classification, pairwise classifications can be used (one SVM is defined by a class against all a convex others, for all optimization classes), which makes it time-consuming especially in the case of a large amount of data.

Huynh and Schiele [67] combined SVM and multiple eigen-spaces approach in order to enhance the standard naive Bayes classifier (see Section 2.3.7) with small numbers of training data. Krause *et al.* [68] considered the recognition of eight common activities using SVM and observed better achievement of frequency-domain features compared to time-domain features.

Doukas and Maglogiannis [69] and Zhang *et al.* [64] applied SVM techniques to discriminate between falls and other activities. A microphone and tri-axial accelerometer were used to identify falls and two activities: walking and running. The recognition rates ranged between 84% and 96%.

#### 2.3.3. Random Forests

Random Forests (RF) [70] consists of a combination of decision-trees. It improves the classification performance of a single-tree classifier by combining the bootstrap aggregating (bagging) method and randomization in the selection of partitioning data nodes in the construction of decision tree. The assignment of a new observation vector to a class is based on a majority vote of the different decisions provided by each tree constituting the forest. However, RF needs huge amount of labeled data to achieve good performances.

In [71], the authors proposed a classification methodology to recognize, using acceleration data, different classes of motions, such as driving a car, being in a train, and walking, by comparing different machine learning techniques (Random Forests, SVM and Naive Bayes). The authors showed that Random Forest algorithm provides the highest average accuracy outperforming the SVMs and the Naive Bayes.

#### 2.3.4. Gaussian Mixture Models

A Gaussian Mixture Model (GMM) is a probabilistic approach, generally used in an unsupervised classification. Unlike standard probabilistic models based on approximating the data by a single Gaussian component density, GMM uses a weighted sum of finite Gaussian component densities. The parameters of GMM (the proportions, the mean vectors and the covariance matrices of the Gaussian components) are estimated using the expectation-maximization (EM) algorithm [72]. Using constructed features for human activity recognition, it is possible to learn separate GMMs for different activities. The data classification can then be performed, by selecting the GMM with the highest posterior probability. One of the drawbacks of this model is that in many cases the GMM does not guarantee the convergence to the global minimum and a particular attention needs to be given to the initialization of the EM algorithm. The GMM has been applied in several studies for human activity recognition as shown in [73].

#### 2.3.5. K-Means

K-means is a well-known unsupervised classification technique that can cluster *n* objects into *k* classes. K-means clustering minimizes the distortion measure the total intra-cluster variance as a cost function. This consists of iteratively finding the cluster centroids, and then assigning the data according to their distance (e.g., Euclidean) to the cluster centroids, until convergence. One of the known limitations of k-means is that it may have poor performance in the case of overlapping clusters (classes) and it does not define a density on the data and cannot therefore measure the uncertainty regarding the data classification, particularly in the overlap regions. As for the use of the K-means for human activity recognition, the reader can refer to [74,75].

#### 2.3.6. Markov Chains and Hidden Markov Models

A Markov chain represents a discrete time stochastic process covering a finite number of states where the current state depends on the previous one [7]. In the case of human activity recognition, each activity is represented with a state. A Markov chain is well adapted to model sequential data and is often used in a more general model that is the Hidden Markov Model (HMM).

The HMM assumes that the observed sequence is governed by a hidden state (activity) sequence. Once the HMM is trained, the most likely sequence of activities can then be determined using the Viterbi algorithm [76]. As in the case of GMM, one of the drawbacks of HMMs is that in many cases this model does not guarantee the convergence to the global minimum and a particular attention needs to be given to the initialization of the EM algorithm known in the context of HMMs as the Baum-Welch algorithm.

In [77], the HMM is used in a two-level classification schema to distinguish different daily living activities. The HMM is trained using the posterior probabilities of the decision stump in order to take advantage of the results from the discriminatively trained classifier (decision stump), as well as to reduce the complexity of the HMM.

HMMs have also been used as a part of unsupervised learning algorithms for human activity recognition studies [78,79,80]. In these studies, an HMM with GMM emission densities was developed using the HMM toolbox [81]. The next section provides a brief summary on other useful techniques that have been used for human activity recognition. 

#### 2.3.7. Other Classification Techniques Used in Activity Recognition

In order to define a given activity, a threshold-based classifier compares different features to a predefined threshold, generally fixed by the user. This approach is sufficient to identify static postures, for instance standing, sitting and lying [82,83]. Postural transitions have been also classified using conventional threshold-based algorithms [13,84]. However, this classification method is sensitive to the chosen thresholds values.

Several studies have shown that combing different threshold rules improves fall detection accuracy. In [85], authors showed that using three threshold-based rules for orientation, angular velocity and angular acceleration, falls can be distinguished with 100% accuracy from everyday living activities. 

Another paradigm for human activity recognition is the one of fuzzy logic methods. Fuzzy logic takes its origin from fuzzy sets theory. It shows a great potential for activity classification problems. However, fuzzy logic needs to employ methods for constructing proper membership functions as well as the combination and the interpretation of fuzzy rules. Besides, only a few studies have shown satisfactory classification accuracies in fall detection. Among the relevant studies that applied fuzzy methods for human activity recognition, one can cite those conducted by Salarian *et al.* [24], Marin-Perianu *et al.* [86] and Masse *et al.* [30]. 

The multilayer perceptron (MLP) [87], is an artificial neural networks with multilayer feed-forward architecture and is in general based on non-linear activations for the hidden units. The MLP minimizes the error function between the estimated and the desired network outputs, which represent the class labels in the classification context. Several studies show that a MLP is efficient in non-linear classification problems, including human activity recognition. The MLP has been applied in several studies for human activity recognition such as [5,7,61].

Another well-known supervised classification technique is the Naive Bayes classifier, which is popular due to its simplicity and ease of implementation. In this approach the input features are assumed to be independent, while the conditional likelihood function of each activity can be expressed as the product of simple probability density functions. For human activity recognition, the naïve Bayes approach shows similar accuracy level when compared to other classification methods. The studies presented in [88,89] showed that sometimes naïve Bayesian approach outperforms other classification approaches, while in [7] the classification accuracy obtained when using naïve Bayes approach is not relatively high. 

The hierarchical classification scheme builds a binary decision structure that consists of numerous consecutive nodes. Relying on the input features, the binary decision is made at each node. The discrimination between activities is achieved based on the decision results. Making decisions at each node requires manual supervision and analysis of the training data making this approach very time consuming. Time-domain features are used in [90] to classify four different activities. Each activity is fully recognized (100%) using accelerometers placed at the chest, wrist, shank and thigh. Similarly, data collected from accelerometers placed at the waist are used in [91] to identify four static and five dynamic activities. In [17,92] a threshold-based hierarchical classification scheme is applied to discriminate different dynamic activities. Moreover, in [92] the performances of the hierarchical approach are compared to those of other standard classification techniques. In [18], authors combined probabilistic methods and signal morphology techniques to generate a classification decision at each node. It is shown that this approach is able to discriminate several human activities. In [15], additional node was used to detect the fall event.

### 2.4. Discussion

It is clear that comparing algorithm performance across different studies is a difficult task for many reasons. This difficulty is mainly related to: (i) the variability in the experimental protocols (the number of recruited subjects, the nature and the number of the recognized activities—ambulation, transportation, daily activities, exercise/fitness—the duration and the order of different activities, *etc*.); (ii) the applicative objectives behind the human activity recognition (monitoring, fall detection, home-based rehabilitation, *etc*.); (iii) the type of sensors used (accelerometers, plantar pressure sensors, gyroscopes) and their attachment to the body (wrist, chest, hip, thigh, necklace); (iv) the performance evaluation criteria (accuracy, F-measure, recall, precision, specificity, *etc*.); (v) the validation procedure (P-fold, leave one out, repeated random sub-sampling, bootstrap, *etc.*). In [14,15,18,20,28,29], one accelerometer was used to recognize activities such as sitting, standing, lying, walking, running, scrubbing, vacuuming, brushing teeth, falling, *etc*. The average classification rate varied from 90.8% and 98.9% and was obtained when using an accelerometer placed at either waist level [15,18,28] or at wrist level [20,29]. In [17,19,23,26], two accelerometers were used to recognize activities such as slow walking, fast walking, and rowing. In most of these studies, the number of activities does not exceed ten activities. A large number of activities were considered in [27] (sixteen activities) and [7] (twelve activities). In those studies, classification rates ranging from 89% to 90.3% were obtained. In others studies, three accelerometers were placed at the thigh, on a necklace and at wrist level [24] and at the level of wrist, chest and hip [22]. Almost similar results than those obtained in [7,27] are achieved in [21,22]. In [8], the authors evaluated the use of a single-accelerometer *versus* a multi-accelerometer to recognize five activities (standing, sitting, lying, walking and transition). A recognition rate of 96.4% was achieved when using multi-accelerometers placed at chest, waist, thigh, and side level. In [24], IMU sensors including accelerometers and gyroscopes, placed at the trunk and the shank levels were used to recognize 14 daily activities of Parkinson’s disease (PD) patients. A sensitivity of 83.8% was achieved in the case of PD patients. In [30], the authors integrate IMU and pressure sensors to improve the recognition rate of daily living activities of stroke patients. A classification rate of 90.4%, was achieved for recognizing basic activities such as lying, sitting, standing, and walking, as well as distinguishing the body elevation such as flat, elevator down, elevator up, stairs down and stairs up.

**Figure 3:**
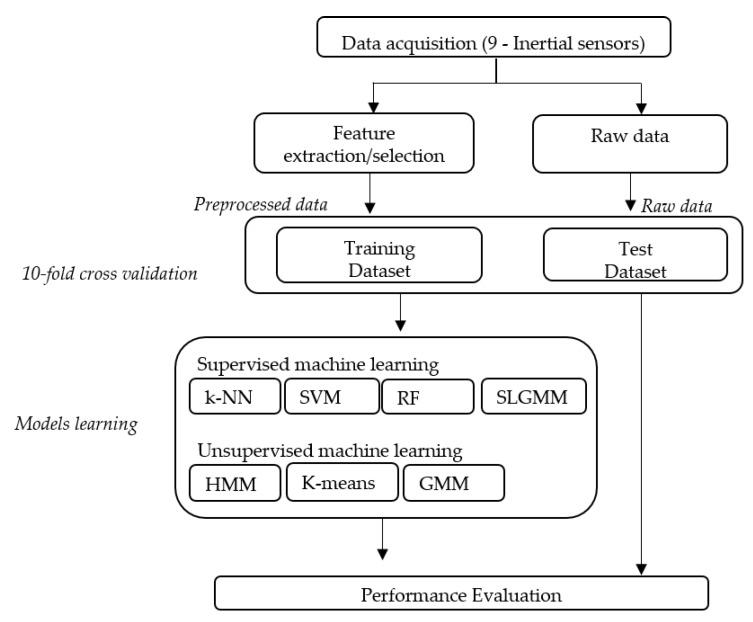
Steps of human activity recognition.

## 3. Methods

In this section, we present the proposed methodology including data acquisition, the used classifiers and the performance evaluation using the 10-fold cross validation method. Figure 3 summarizes the different steps of the adopted approach.

### 3.1. Data Acquisition

**Figure 4:**
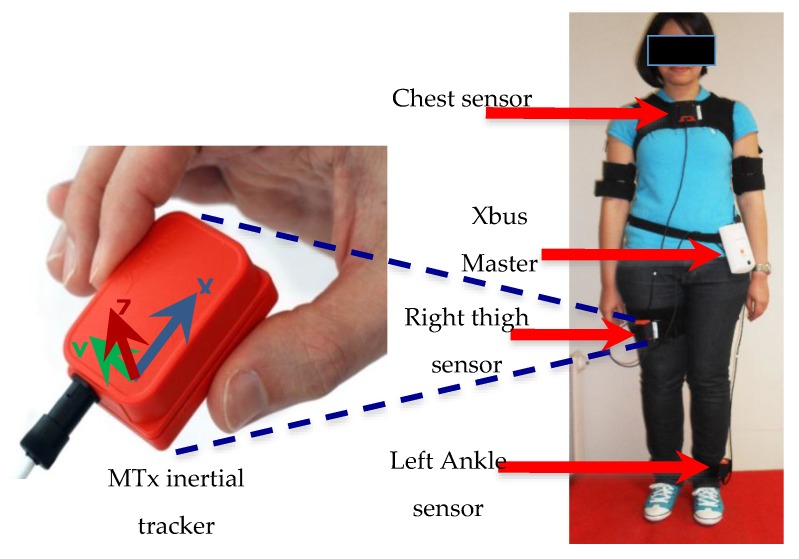
MTx-Xbus inertial tracker and sensors placement.

In this study, human activities are estimated using the Xbus Kit from Xsens (Enschede, Netherlands) which enables ambulatory measurement of the human motion. It consists of a portable system that incorporates an Xbus Master and three MTx inertial units that are placed on the chest, the right thigh and the left ankle of the subject see Figure 4. Each MTx unit is equipped with a tri-axial accelerometer to measure the 3D acceleration, a gyroscope to measure the 3D angular velocity, and a magnetometer to measure the local Earth magnetic field vector. The MTx units located at the chest, thigh and ankle levels are connected in series with the Xbus Master. The collected data are transmitted from the Xbus Master to the host PC using a Bluetooth link. The power consumption of five MTx units is 2.7 W when using the Bluetooth communication protocol. Four NiMH AA 2500 mAh rechargeable batteries are included for remote use. The maximum duration of measurements is 3 h for five MTx using the Bluetooth communication protocol. The sampling frequency of this system is set to 25 Hz, which is sufficient to measure daily human physical activities [39]**.** A fundamental problem in human motion analysis based on the use of IMUs is the alignment between the IMUs’ local coordinate axes and a physiologically meaningful axis. Actually a small tilt or misplacement of the sensor would result in large variation on the measured data. It should be noted that some research works completely ignore this problem by assuming that the IMUs can be mounted precisely in a predefined orientation towards the joint [93,94]. In this study, the IMUs sensors were very securely attached to the participant’s body using special straps provided by the Xsens Company. These straps avoid any relative movements and misalignments between the wearable sensors and the associated body member. Moreover, the sensor-to-segment mounting orientation and position are characterized by the local coordinates of the joint axis and the joint position, respectively. In this study an external operator has manually verified both quantities prior to each experimentation test. 

Data were collected at the LISSI Lab/University of Paris-Est Creteil (UPEC). Six healthy subjects with different profiles (mean age: 26 years old, mean weight: 65 kg) participated in the experiments. The subjects were given instructions to perform activities in their own way without specific constraints. Each subject conducted a total of twelve activities. The data acquisition was performed in the office environment over a period of about 30 min. The different activities and their descriptions are given in Table 2. The acquired data were manually labeled by an independent operator.

**Table 2:** List of the selected activities (A1…A12).

Activity Reference	Description of Activity
A1	Stair descent
A2	Standing
A3	Sitting down
A4	Sitting
A5	From sitting to sitting on the ground
A6	Sitting on the ground
A7	Lying down
A8	Lying
A9	From lying to sitting on the ground
A10	Standing up
A11	Walking
A12	Stair ascent

**Figure 5:**
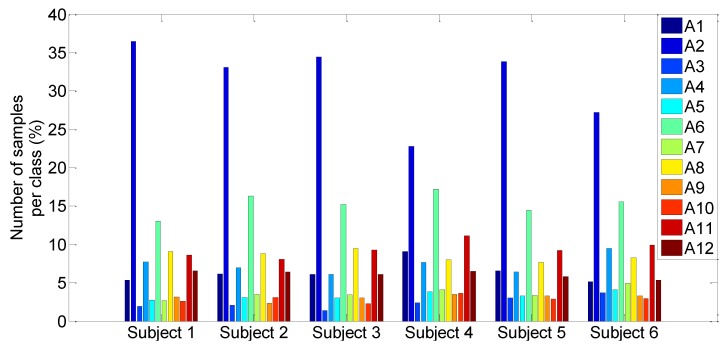
Representation of the number of samples in each class for each sequence.

The dataset is composed of six tests where each one was performed according to the following sequential activities order: A2 A1 A2 A3 A4 A5 A6 A7 A8 A9 A6 A10 A2 A11 A2 A12. Figure 5 shows the number of samples in each class (each activity corresponds to a class) for each sequence. One can note that the different classes are not equally distributed. The transition activities A3, A5, A7, A9, A6 and A10 are weakly represented compared to other activities. We also note that the majority of the sequences are composed of some sort of standing activity (about 32% of the full dataset).

As mentioned earlier, two different (supervised and unsupervised) techniques were used to recognize the twelve human activities. Four supervised machines learning techniques, that are, k-NN, SVM, SLGMM and RF, and three unsupervised machine learning techniques, that are, K-means, GMM and HMMs are compared in terms of performances with respect to the human activity recognition rate. Two cases were considered in terms of input data:

- Raw data
- Feature set extracted/selected from raw data.

### 3.2. Classifiers

#### 3.2.1. Supervised Machine Learning Techniques

- In this study, the LIBSVM toolbox [95] was used to implement a nonlinear SVM model with a radial basis function kernel. The hyper-parameters C and Ɣ are estimated using a grid search method. The optimal values are C = 2 and Ɣ = −5.
- In the case of the RFs algorithm, the only parameter to tune is the number of trees, which is adjusted by varying the number of trees from to 1 to 100 and determining the one providing the best accuracy rate. The best number of trees is 20.
- For the SLGMMs, a mixture of 12 diagonal Gaussians is used. The proportions, the mean vectors and the covariance matrix of the Gaussian components are estimated during the training phase using an appropriate EM algorithm.
- In the case of K-NN method, as the only parameter to tune is K, varying K from 1 to 20 leads to an optimal value of K=1 for the best accuracy.

#### 3.2.2. Unsupervised Machine Learning Techniques

- In this study, HMM with GMM emission probabilities were developed using the HMM toolbox [81]. However, two hyper-parameters were tuned: the number of states and the number of mixtures. First, as the dataset consist of twelve activities, the number of states was set to 12 with ergodic topology. Then, number of mixtures was varying from 1 to 4. Based on the best accuracy rate, the states were modeled using a mixture of 2 diagonal Gaussians.
- In the case of the K-means algorithm, the only parameter to estimate is the number of clusters that corresponds to the number of activities (k=12).
- In the case of the GMM algorithm, as in the case of the K-means algorithm, the only parameter to estimate is the number of mixture, which corresponds to the number of activities. A mixture of 12 diagonal Gaussians was used.

In this study, a 10-fold cross validation procedure was used to create the training set and the test set. In the case of supervised approaches, the models were trained using the training set. In the test step, the estimated classes were compared to the true classes in order to compute the classification error rates. In the case of the unsupervised approaches, the models were trained using only the raw data without considering the labels. The labels were only used to evaluate the classification performances. In the test step, the classifiers outputs (estimated labels) were matched with the true labels in order to evaluate the classifier performances.

### 3.3. Evaluation

The accuracy measure is used to evaluate the classifiers performances. In fact, this metric measures the proportion of correctly classified examples. In the case of binary classification, the accuracy can be expressed as follows:

(7) $$\text{Accuracy}=\frac{T_{p}+T_{n}}{T_{p}+T_{n}+F_{p}+F_{n}}$$

where $T_{n}$ (true negatives) represents the correct classifications of negative examples, $T_{p}$ (true positives) represents the correct classifications of positive examples. $F_{n}$ (false negatives) and $F_{p}$ (false positives) represent, respectively the positive examples incorrectly classified into the negative classes and the negative examples incorrectly classified into the positive classes. 

The accuracy measure does not take into account the unbalanced datasets. In this case, the accuracy is particularly biased to favor the majority classes. In this study, the class proportions are not well balanced since the proportion of transitions activities samples is too small (Figure 5). Thus the following evaluation criteria are considered: the average of the accuracy rate (R) and its standard deviation (std), F-measure, recall, precision and specificity. 

The F-measure is defined as the combination of two criteria, the precision and the recall, which are defined as follows:

(8) $$\text{precision}=\frac{T_{p}}{T_{p}+F_{p}}$$

(9) $$\text{recall }=\frac{T_{p}}{T_{p}+F_{n}}$$

The F-measure is calculated as follows:

(10) $$\text{F}\_\text{measure }=\frac{\left(1+\beta^{2}\right).\text{recall}.\text{precision }}{\beta^{2}\text{ recall}+\text{precision}}$$

where β is a weighting factor that controls the degree of importance of recall/precision. This parameter is a positive real number. In this study, β is set to 1 to give the same importance to both recall and precision.

The specificity (SPC) is also used to evaluate the performances of the different algorithms and is calculated as follows:

(11) $$\text{specificity}=\frac{T_{n}}{T_{n}+F_{p}}$$

## 4. Experimental Results

In this section, we review and compare the performances of the standard supervised and unsupervised machine learning approaches to recognize the daily living activities presented in the previous section. This comparison highlights the different algorithm performances in terms of average accuracy rate (R) and its standard deviation (std), F-measure, recall, precision and specificity. In this comparative study, two cases are considered: 

### 4.1. Case 1: Raw Data

The results obtained in the case of raw data are given in Table 3 and Table 4. Table 3 summarizes the performance results obtained when using the supervised approaches. It can be observed that the correct classification rates obtained with different techniques are all higher than 84%. The k-NN algorithm gives the best results in terms of global correct classification rate, F-measure, recall, and precision, followed by RF, then SVM and at finally the SLGMM algorithm gives relatively the worst results.

**Table 3:** Performances of the supervised algorithms using raw data.

	Accuracy ± std	F-measure	Recall	Precision	Specificity
k-NN (%)	96.53 ± 0.20	94.60	94.57	94.62	99.67
RF (%)	94.89 ± 0.57	82.87	82.28	83.46	99.43
SVM (%)	94.22 ± 0.28	90.66	90.98	90.33	99.56
SLGMM (%)	84.54 ± 0.30	69.94	69.99	69.88	98.39

**Table 4:** Performance results of the unsupervised algorithms using raw data.

	Accuracy ± std	F-measure	Recall	Precision	Specificity
HMM (%)	**80.00 ± 2.10**	**67.67**	**65.02**	**66.15**	**97.68**
K-means (%)	68.42 ± 5.05	49.89	48.67	48.55	**93.21**
GMM (%)	73.60 ± 2.32	57.68	57.54	58.82	**96.45**

Table 4 summarizes the results obtained when using the different unsupervised learning approaches. Compared to the unsupervised classifiers K-means and GMM, the HMM approach gives the best results in terms of global correct classification rate, F-measure, recall, and precision. These results can be explained by the fact that the HMM approach takes into account the temporal aspect of the data used in this study. Table 3 and Table 4 show that supervised approaches outperform unsupervised approaches. However, unsupervised approaches show very encouraging results mainly in the case of HMM. These performances are obtained without any labeling that is time consuming. 

Furthermore, the used supervised classifiers need labeled data in the training model phase. In addition, these methods do not take into account the sequential dimension of the data in their model formulation. Indeed, the dependencies between the activities are neglected in the learning phase as well as in the testing phases. Moreover, it is worth pointing out that in the case of k-NN based approach, a considerable computation time is required due to the fact that assigning a new sample to a class require a computation time as many distances as there are examples in the dataset.

In order to identify the patterns that are difficult to recognize, the global confusion matrix are given in Table 5 and Table 6 in the case of k-NN and HMM, respectively. One can observe that confusions in most cases, occur between transition activities such as (A_9_, A_7_) and dynamic activities such as (A_1_, A_11_), (A_1_, A_12_) and (A_11_, A_12_). These confusions are more important in the case of HMM. One can also observe that the basic activities such as A_2_, A_4_, A_8_ are easier to recognize than transition activities such as A_3_, A_5_ and A_7_.

**Table 5:** Global confusion matrix obtained with k-NN using raw data.

						Obtained	Classes						
		**A_1_**	**A_2_**	**A_3_**	**A_4_**	**A_5_**	**A_6_**	**A_7_**	**A_8_**	**A_9_**	**A_10_**	**A_11_**	**A_12_**
	**A_1_**	88.98	0.41	0.04	0	0.04	0	0	0	0	0.78	4.34	5.41
	**A_2_**	0.40	98.52	0.08	0	0	0	0	0	0	0.21	0.56	0.23
	**A_3_**	0.21	0.64	95.73	0.53	0.64	0	0	0	0	0.96	0.85	0.43
	**A_4_**	0	0	0.77	98.92	0.31	0	0	0	0	0	0	0
**True**	**A_5_**	0.08	0	0.55	0.16	97.98	0.47	0.08	0	0.16	0.55	0	0
**Classes**	**A_6_**	0	0	0	0	0.22	99.41	0.03	0	0.25	0.08	0	0
	**A_7_**	0	0	0	0	0.22	0.15	95.71	1.53	2.33	0.07	0	0
	**A_8_**	0	0	0	0	0	0	1.58	97.62	0.80	0	0	0
	**A_9_**	0	0	0	0	0.25	0.34	3.96	0.67	94.44	0.34	0	0
	**A_10_**	1.58	0.46	0.19	0	0.65	0.28	0	0	0.19	94.07	0.93	1.67
	**A_11_**	4.07	0.41	0.03	0	0	0	0	0	0	0.55	92.57	2.37
	**A_12_**	5.05	0.43	0	0	0	0	0	0	0	1.03	3.08	90.42

**Table 6:** Global confusion matrix obtained with HMM using raw data.

						Obtained	Classes						
		**A_1_**	**A_2_**	**A_3_**	**A_4_**	**A_5_**	**A_6_**	**A_7_**	**A_8_**	**A_9_**	**A_10_**	**A_11_**	**A_12_**
	**A_1_**	55.33	1.70	1.08	0	0.62	0	0	0	0	3.19	23.52	14.57
	**A_2_**	2.83	86.22	0.47	0	0	0	0	0	0	1.50	6.97	2.01
	**A_3_**	0.12	0	39.86	32.82	12.53	0	0	0	0	10.62	0.24	3.82
	**A_4_**	0.10	0	9.58	87.21	3.11	0	0	0	0	0	0	0
**True**	**A_5_**	0.67	0	7.20	0.29	73.61	0.10	1.06	0	1.44	15.55	0	0.10
**Classes**	**A_6_**	0	0	0	0	3.15	91.63	0.88	0	2.18	2.16	0	0
	**A_7_**	0	0	0	0	2.24	0.50	29.74	35.33	27.95	4.25	0	0
	**A_8_**	0	0	0	0	0	0	13.14	81.38	5.48		0	0
	**A_9_**	0	0	0	0	2.13	0	37.03	16.70	33.75	10.39	0	0
	**A_10_**	0	0	0	0	9.20	0	0	0	1.15	89.66	0	0
	**A_11_**	19.59	1.38	2.53	0	0	0	0	0	0	2.38	56.95	17.17
	**A_12_**	16.65	0	3.72	0	2.44	0	0	0	0	5.75	11.10	60.34

### 4.2. Case 2: Feature Set Extracted/Selected from Raw Data

In order to improve the results presented above a preprocessing step consisting of features extraction and selection is performed. Nine accelerometrics signals are acquired from three MTx IMUs and for each signal; the following time and frequency domain features are calculated:

- Eleven time-domain features are extracted, namely: mean, variance, median, interquartile rang, skewedness, kurtosis, root mean square, zero crossing, peak to peak, crest factor and rang.
- Six frequency-domain features are extracted, namely: DC component in FFT spectrum, energy spectrum, entropy spectrum, sum of the wavelet coefficients, squared sum of the wavelet coefficients and energy of the wavelet coefficients.

In addition, the correlation coefficients of mean and variance of the norm of each acceleration signal are calculated. Thus, a set of nine correlation coefficients, six means and variances of the norm of each acceleration signal are calculated.

**Figure 6:**
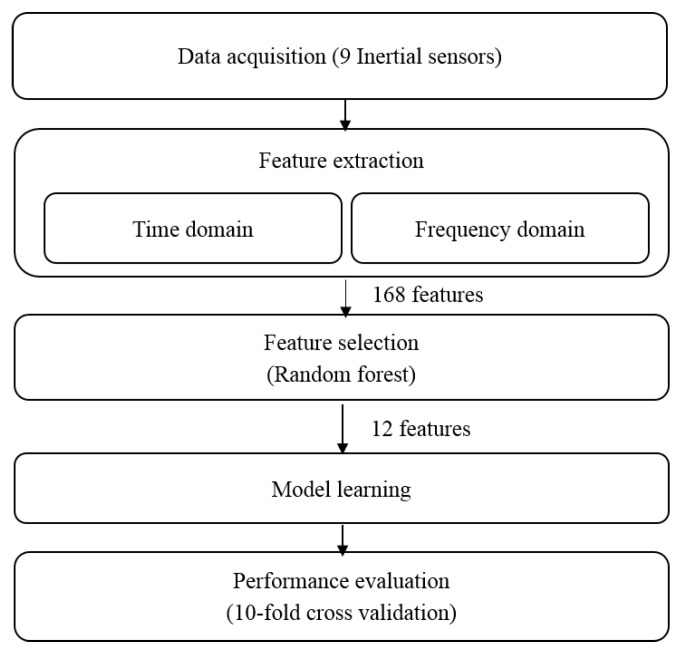
Steps of the activity recognition process using features’ extraction and selection.

A total of 168 characteristics are calculated for each sliding window with a size of 25 samples (1 s each) with 80% overlap. The window size is chosen to ensure the statistical significance of the calculated features. The choice of the window-overlapping rate is done in order to guarantee satisfactory characterization of the transitional activities, which are ephemeral. In our case, the transitional activities take about 2 s, thus using windows of 25 samples without overlapping leads to the extraction of features with just two samples, which are not sufficient to correctly characterize these transitions. Following the feature extraction step, a process is performed to find a minimal subset of features that are necessary and sufficient to adequately characterize the different activities. As described above, finding the best subset among all features is carried out during the feature selection procedure. In this study, a wrapper approach based on random forest feature selection algorithm [70] is used to select the best features among the extracted ones. This algorithm reorders the features according to their relevance percentage. A set of 12 features representing more than 80% of relevance, are selected as the classifiers inputs. Figure 6 describes the different steps of the activity recognition process using the selected features.

The results obtained using the supervised approaches with extracted/selected features are reported in Table 7. The correct classification rates obtained with the different techniques are greater than 85%. Similarly to the case of raw data, k-NN algorithm gives the best results in terms of correct classification rate and its standard derivation, F-measure, recall, and precision, followed by RF, then SVM and finally SLGMM. As it can be shown, a significant improvement for some algorithms can be observed (an average improvement of 3% with a slight reduction of *std* is observed for k-NN and RF). In the case of SVM and SLGMM, a slight improvement about 1% can be observed on the correct rate. Regarding F-measure, recall and precision, an average improvement of 10% and 4% are observed for SVM and SLGMM, respectively. The results obtained using unsupervised machine learning techniques in the case of selected features are reported in Table 8. These results show an improvement in terms of correct classification rate, F-measure, recall and precision. Besides, in the case of HMM, an improvement of 3% of global rate with slight reduction of *std* (about 0.8%) can be observed, while F-measure, recall and precision increase by about 3%, respectively. Improvements can be also noted for the performances of the GMM. In the case of K-means, an improvement of 4.53% and 3.53% of global correct rate classification and recall, respectively along with a decrease of 3% of *std* can be observed. A slight improvement of about 0.4% and 2.67% can also be observed on F-measure and precision respectively. Finally, even though improvements in terms of performances are observed when using the selected features as input for the different algorithms, the feature extraction/selection step requires implementing additional models and routines, to extract and select optimal features. Moreover, the feature extraction process needs additional computational time, which can be challengeable for real time applications.

**Table 7:** Performances of the supervised algorithms using extracted features.

	Accuracy ± std	F-Measure	Recall	Precision	Specificity
k-NN (%)	**99.25 ± 0.17**	**98.85**	**98.85**	**98.85**	**99.96**
RF (%)	98.95 ± 0.09	98.27	98.24	98.25	99.90
SVM (%)	95.55 ± 0.30	93.02	93.15	92.90	99.92
SLGMM (%)	85.05 ± 0.57	73.44	74.44	73.61	99.88

**Table 8:** Performances of the unsupervised algorithms using extracted features.

	Accuracy ± std	F-Measure	Recall	Precision	Specificity
HMM (%)	**83.89 ± 1.30**	**69.19**	**68.27**	**67.74**	**98.38**
K-means (%)	72.95 ± 2.80	50.29	52.20	51.22	97.04
GMM (%)	75.60 ± 1.25	65.00	66.29	64.30	97.12

Table 9 and Table 10 represent confusion matrix obtained with k-NN and HMM using selected features. 

**Table 9:** Global confusion matrix obtained with k-NN using selected features.

						Obtained	Classes						
		A_1_	A_2_	A_3_	A_4_	A_5_	A_6_	A_7_	A_8_	A_9_	A_10_	A_11_	A_12_
	A_1_	99.00	0.32	0	0	0	0	0	0	0	0.08	0.48	0.12
	A_2_	0.06	99.75	0.04	0	0	0	0	0	0	0.03	0.07	0.04
	A_3_	0	0.43	99.15	0.43	0	0	0	0	0	0	0	0
	A_4_	0	0	0.11	99.79	0.11	0	0	0	0	0	0	0
**True**	A_5_	0	0	0	0.23	99.38	0.23	0	0	0.08	0.08	0	0
**Classes**	A_6_	0	0	0	0	0.07	99.78	0.07		0.03	0.05	0	0
	A_7_	0	0	0	0	0	0.21	99.65	0.14	0	0	0	0
	A_8_	0	0	0	0	0		0.15	99.79	0.06		0	0
	A_9_	0	0	0	0	0.08	0.17		0.33	99.42	0	0	0
	A_10_	0.35	0.18	0	0	0.09	0.09	0	0	0	99.20	0.09	
	A_11_	0.22	0.17	0	0	0	0	0	0	0	0	99.34	0.28
	A_12_	0.08	0.17	0	0	0	0	0	0	0	0.04	0.25	99.45

**Table 10:** Global confusion matrix obtained with HMM using selected features.

						Obtained	Classes						
		A_1_	A_2_	A_3_	A_4_	A_5_	A_6_	A_7_	A_8_	A_9_	A_10_	A_11_	A_12_
	A_1_	57.74	0.06	0.43	0	0.31	0	0	0	0	4.07	20.17	17.21
	A_2_	1.36	94.66	0.31	0	0	0	0	0	0	0.89	1.98	0.80
	A_3_	3.82	0	55.30	5.69	15.42	0	0	0	0	1.64	4.91	13.24
	A_4_	0	0	2.85	96.31	0.83	0	0	0	0	0	0	0
**True**	A_5_	2.05	0	1.80	0.66	71.62	4.35	2.21	0	5.50	11.48	0	0.33
**Classes**	A_6_	0	0	0	0	1.39	97.09	0.30	0	0.94	0.28	0	0
	A_7_	0	0	0	0	1.54	0	59.91	4.25	32.30	1.99	0	0
	A_8_	0	0	0	0	0	0	3.30	94.69	2.01		0	0
	A_9_	0	0	0	0	4.02	1.75	32.68	0.10	50.41	11.03	0	0
	A_10_	13.56	0	1.51	0	6.44	0	1.92	0	2.19	60.68	7.12	6.58
	A_11_	19.87	4.45	1.50	0	0	0	0	0	0	3.45	57.02	13.73
	A_12_	16.37	0.17	0	0	0.34	0	0	0	0	1.90	17.26	63.97

The same observations can be made as in the case of using raw data as input classifiers. However, a considerable improvement can be observed compared to those obtained when using raw data. This can be explained by the fact that the extracted/selected features characterize well the different human activities. As stated above, comparing different algorithms’ performance across different studies is a difficult task for many reasons, such as the experimental protocol differences, the applicative objectives behind human activities recognition, the type of sensors used and their attachment to the body, the performance evaluation and validation and the nature/number of the recognized activities. The obtained results in terms of accuracy rate are almost similar to those obtained in related studies (e.g., [7,25,26,27]). For example in the case of k-NN, accuracy rates of 95.8% and 98.7% were obtained in [7] and [5] respectively. In the case of SVM, SLGMM and RF, similar performances were obtained in [5,7,70,73,75]. In the case of unsupervised based classifiers such as K-means, GMM, and HMM, the accuracy rates, range from 60.2% to 84% ([7,75,78,79,80]). It should be noted that the studies using feature extraction/selection as classifiers inputs have shown better performances, e.g., [5,62,63]. These results are also confirmed in this study.

## 5. Conclusions and Future Work

We have presented a review of different classification techniques that were used to recognize human activities from wearable inertial sensor data. This paper describes the whole structure of the recognition detection process, from data acquisition to classification. Issues related to wearable sensor’s properties and placement on the human body are addressed first. Feature computation, selection and extraction processes are shown, followed by a literature review comparison between various supervised and unsupervised learning approaches used for classifying of daily human activities. Finally, we presented a comparative study using well-known supervised and unsupervised based approaches (k-Nearest Neighbors, Gaussian Mixture Models in both cases supervised and unsupervised approaches, Support Vector Machines, Random Forest, k-means and Hidden Markov Models) applied on a real dataset. Both, raw data and extracted/selected features are used as inputs for the classifiers. The different classification approaches are compared in terms of the recognition of twelve activities (including static, dynamic and transition activities) using data from three MTx inertial IMUs placed at the chest, the right thigh and the left ankle. 

The supervised approaches, when using raw data or extracted/selected features, are more accurate compared to unsupervised approaches, yet the latter are more computationally efficient and do not require any labels (unsupervised classification techniques are able to directly create models from unlabeled data). 

The results obtained with the real dataset show the effectiveness of the k-NN approach, which gives the best results compared to the other methods. RF and SVM give almost the same results and slightly better in the case of RF, especially when using extracted/selected features. The SLGMM-based algorithm gives the lowest results in the case of supervised approaches. In the case of unsupervised approaches, HMM gives the best results, followed by GMM and K-means. The main advantage of the HMMs with regards to other techniques is that the statistical model used in the HMMs includes both sequential aspect and temporal evolution of the data. Except for HMMs, the other algorithms treat the data as several realizations in the multidimensional space without taking into the consideration possible dependencies between the activities. 

We have also seen that the extracted/selected features improve the classification accuracy at the expense of computation time that can be penalizing, in particular for real time applications. This work can be extended in several directions: the combination of several classifiers constitutes a promising approach as many classifiers applied to the same dataset have the potential to generate different decision boundaries, which are able to display different patterns. Thus, merging the classification techniques would give complementary decisions and advance the accuracy level. As stated above, the different machine learning methods in both supervised and unsupervised contexts, were evaluated with six participants. Future steps involve expansion of the dataset by adding further participants in general and, in particular, elderly subjects. Another point that deserves to be adequately and further assessed is related to the wearer’s privacy and the acceptability of the wearable sensors. Yet the use of inertial sensors less invade the wearer privacy compared to the use of cameras, few studies in the literature have investigated the acceptability of these sensors in close contact with the users.
